# Interventions for acute stroke management in Africa: a systematic review of the evidence

**DOI:** 10.1186/s13643-017-0594-4

**Published:** 2017-10-24

**Authors:** Leonard Baatiema, Carina K. Y. Chan, Adem Sav, Shawn Somerset

**Affiliations:** 1 Regional Institute for Population Studies, University of Ghana, Legon, Accra Ghana; 2 School of Allied Health, Faculty of Health Sciences, Australian Catholic University, Brisbane, Australia; 3 School of Psychology, Faculty of Health Sciences, Australian Catholic University, Brisbane, Australia

**Keywords:** Stroke, Organised care, Stroke service, Africa, Evidence-based practice, Implementation

## Abstract

**Background:**

The past decades have witnessed a rapid evolution of research on evidence-based acute stroke care interventions worldwide. Nonetheless, the evidence-to-practice gap in acute stroke care remains variable with slow and inconsistent uptake in low-middle income countries (LMICs). This review aims to identify and compare evidence-based acute stroke management interventions with alternative care on overall patient mortality and morbidity outcomes, functional independence, and length of hospital stay across Africa.

**Methods:**

This review was conducted according to the Preferred Reporting Items for Systematic Reviews and Meta-Analyses (PRISMA) guideline. An electronic search was conducted in six databases comprising MEDLINE, Embase, Cumulative Index to Nursing and Allied Health Literature (CINAHL), Web of Science, Academic Search Complete and Cochrane Library for experimental and non-experimental studies. Eligible studies were abstracted into evidence tables and their methodological quality appraised using the Joanna Briggs Institute checklist. Data were analysed and presented narratively with reference to observed differences in patient outcomes, reporting *p* values and confidence intervals for any possible relationship.

**Results:**

Initially, 1896 articles were identified and 37 fully screened. Four non-experimental studies (three cohort and one case series studies) were included in the final review. One study focused on the clinical efficacy of a stroke unit whilst the remaining three reported on thrombolytic therapy. The results demonstrated a reduction in patient deaths attributed to stroke unit care and thrombolytic therapy. Thrombolytic therapy was also associated with reductions in symptomatic intracerebral haemorrhage (SICH). However, the limited eligible studies and methodological limitations compromised definitive conclusions on the extent of and level of efficacy of evidence-based acute stroke care interventions across Africa.

**Conclusion:**

Evidence from this review confirms the widespread assertion of low applicability and uptake of evidence-based acute stroke care in LMICs. Despite the limited eligible studies, the overall positive patient outcomes following such interventions demonstrate the applicability and value of evidence-based acute stroke care interventions in Africa. Health policy attention is thus required to ensure widespread applicability of such interventions for improved patients’ outcomes. The review findings also emphasises the need for further research to unravel the reasons for low uptake.

**Systematic review registration:**

PROSPERO CRD42016051566

**Electronic supplementary material:**

The online version of this article (10.1186/s13643-017-0594-4) contains supplementary material, which is available to authorized users.

## Background

Stroke is a major public health concern worldwide. Despite major advances in medical research and technology for acute stroke care treatment and management, in 2013, it accounted for about 6.5 million deaths and 25.7 million stroke survivors were burdened with multiple debilitating impairments worldwide [1]. However, the distribution of the global burden of stroke is uneven, with low-middle income countries (LMIC), especially those in Africa being disproportionately affected. In Africa, this burden is further accentuated by the increasing prevalence of hypertension [2–5]. This notwithstanding, the nature of acute stroke care is often poor due to the fact that the application of evidence-based acute stroke care interventions for optimal patient outcomes in such countries remains inadequate [6–9]. Evidence-based acute stroke care interventions in this context apply to all scientifically proven therapies, treatment procedures or service intervention for the provision of acute stroke care in clinical settings for optimal patient outcomes. In the context of Africa, there is low allocation to the national health budgets [10]. Compounded to this is the fact that the African continent is currently facing an epidemiological transition, precipitated by rapid unplanned urbanisation, ageing population and increasing modifiable risk factors for non-communicable diseases [11]. Yet prioritising the delivery of standardised care for acute stroke and other non-communicable diseases (NCDs) remain low [12, 13]. This makes it extremely difficult for most healthcare systems to provide standardised care.

Internationally, among the range of diverse acute stroke care interventions and services, four are recommended by most stroke experts as the most effective front-line interventions to significantly reduce stroke-related mortality and morbidity [14, 15]. These interventions comprise having a specialised stroke unit care [16], thrombolytic therapy through tissue plasminogen activator (t-PA) for acute ischemic stroke within 4.5 h of a stroke [17–20], aspirin therapy for acute ischaemic stroke within 48 h of a stroke [21] and decompressive surgery within 48 h of an acute stroke [22]. In recent times, endovascular therapy has also shown tremendous promise for improved neurological outcomes following a stroke [23, 24]. The stroke unit care, for example, has been distinguished as a core component of modern stroke services given its proven benefits to stroke patients in general [16] and the cost-effectiveness of such care [25–27]. A stroke unit is a designated ward where a multidisciplinary team specialised in stroke treatment, and management provides exclusive care for acute stroke patients [16]. The multidisciplinary team includes medical, nursing and therapy/allied health staff, comprising specifically of physiotherapists, speech therapists, occupational therapists, pharmacists, dietitians, radiologists, clinical psychologists, and social workers [28].

More importantly, existing evidence uptake of such interventions is much lower in LMIC such as Africa [7, 9, 29], despite such countries bearing much of the global stroke burden. The provision of care for stroke patients within such resource-poor settings is often poor and fragmented [8, 30, 31] and less likely to follow evidence-based recommendations due to limited resources [32]. For example, a recent review on the global uptake of thrombolytic therapy revealed only 19% uptake in LMIC compared to 50% high-income countries (HIC) [7]. Evidence from the UK estimated 82% of patients receive care in a stroke unit [23] and another 86% in Sweden [25]. Such disparities in uptake apparently warrant global policy actions to ameliorate this situation given that LMICs bear a larger share of the global burden of stroke and yet have limited access to the best interventions for optimal patient care. Previous research has reported that barriers such as limited health policy priority, patient, health professionals’ and other organisational context factors potentially underpin the currently low uptake of evidence-based interventions for acute stroke care in Africa [33].

Given indications of variable and poor nature of acute stroke services in Africa, evidently manifested in high case fatality rates of about 40% in Ghana [34, 35] and 70% in Mozambique [36], it is important to understand the exact nature of acute stroke care interventions in this particular region of the world. However, the extent to which evidence-based acute stroke management interventions are used within the African region specifically is not well understood, and so there is insufficient knowledge on the forms of acute stroke care interventions and whether such interventions result in optimal patient outcomes. Although, some reviews have been conducted on the use of these acute stroke care interventions in LMICs [7, 31], these works did not focus exclusively on Africa. This study aims to identify and compare four recommended acute stroke management interventions (stroke unit, thrombolytic therapy, aspirin and decompressive surgery) with alternative care on overall patient mortality and morbidity outcomes, functional independence and length of hospital stay across hospital settings in Africa. A synthesis of this evidence will address the current knowledge gap on the application of evidence-based acute stroke management and potentially help formulate strategies to strengthen the clinical capacity of the current healthcare system to improve uptake of current interventions in Africa.

## Methods

This review was guided by the standardised Preferred Reporting Items for Systematic reviews and Meta-Analyses (PRISMA) approach [37]. The review protocol was registered (PROSPERO 2016: CRD42016051566).

### Eligibility criteria

#### Study design

Studies for this review comprised randomised control trials (RCTs), quasi-randomised trials, non-randomised clinical studies, quasi-experimental studies and reporting on acute stroke care. To ensure inclusion of all potentially relevant studies, prospective and retrospective cohort studies, case-control studies, before-and-after studies and analytical cross-sectional studies were also considered. Included studies also reported patient outcomes after the use of an in-patient stroke treatment and management intervention or therapy. To qualify for inclusion, eligible studies also reported patients’ baseline characteristics and duration of follow-up. Editorials or opinion pieces related to the subject of the review were excluded.

#### Participants

This review considered studies on adult stroke patients 18 years and older, of either sex. Studies reporting patients diagnosed and treated for transient ischaemic attack were excluded. Studies which included a mix of patients with stroke and other health conditions were also excluded.

#### Interventions

Studies evaluating the efficacy of acute stroke care interventions were included. The interventions of interest included the use of aspirin, thrombolytic therapy and haemicraniectomy or decompressive surgery. Studies which reported on acute stroke outcomes following multidisciplinary stroke team care in a stroke unit were also included. Additionally, secondary interventions of interest such as endovascular therapy were included. The comparators of interest included normal care, conventional care or no other treatment.

#### Outcome measures

Study outcomes were categorised into two: primary and secondary. The primary outcomes of interest were interventions reporting on in-patient deaths (mortality outcomes), length of hospital stay, functional independence and morbidity outcomes such as asymptomatic intracranial haemorrhage and extracranial haemorrhage. Secondary outcomes included patient access to the following acute stroke care services: magnetic resonance imaging, computed tomographic scan, electrocardiogram (ECG) and carotid doppler services.

### Search strategy and selection criteria

An electronic search of six databases comprising MEDLINE, Embase, CINAHL, Academic Search Complete, Web of Science and Cochrane Library was conducted. Databases were searched individually to ensure all relevant studies were considered. Other sources such as Google Scholar, African Journals Online and African Index Medicus were also searched. In addition, reference lists and bibliographies from eligible studies were screened manually for further eligible studies. The year limit for searches was opened up to November 2016 and only studies published in English or French were considered. Finally, studies had to be conducted in an African country hospital setting. For search terms, an initial scoping of literature was undertaken to identify keywords, subject-specific terms or MeSH terms related to stroke and the acute stroke care interventions. An example of the search strategy in MEDLINE database employed in the search process is provided (Additional file 1).

#### Study selection and data extraction

Selection and extraction of potential studies were conducted through a four-step process. First, one author (LB) screened and retrieved all potential studies and consequently imported them into a reference manager (EndNote). In the second stage, the remaining studies were screened by two authors (LB and SS) for eligibility on the basis of title and abstract relevance. The third stage involved cross-checking of studies eligible for full-text screening by a third author (AS) in order to minimise selection bias. The final stage involved full-text screening to select studies meeting the inclusion criteria or considered potentially relevant by one author (LB), and this was double checked by another author (SS). Using a standardised pre-designed data extraction form, all eligible studies were extracted according to author(s), year of publication, country of study origin, study aim, population characteristics and sample size, level of evidence, intervention type, comparator, study duration, outcomes of interest and key findings.

#### Assessment of methodological quality

To minimise bias and improve the strength of evidence, the quality of each included study was first assessed independently by one author (LB) applying the Joanna Briggs Institute quality appraisal tool for assessing risk of bias in observational cohort studies and case series [38]. This was verified by other authors (CKYC and SS). A joint discussion was conducted to achieve consensus where differences emerged during quality assessment. Assessment of study quality for risk of bias was conducted based on how participants were selected, sampling approach, representativeness of sample, study design, assessment of exposure, adequacy of case definition and selection of controls exposure for all study types. In classifying the evidence levels for each of the eligible studies, the Oxford Centre for Evidence-Based Management framework was employed [39]. This is an established and widely applied framework in classifying the evidence levels of clinical experimental and non-experimental study designs based on the best available scientific evidence.

#### Data synthesis

This review followed the narrative synthesis framework by Popay et al. [40] in conducting data synthesis in systematic reviews. To minimise heterogeneity effects resulting from the diverse reported study designs, extracted data were managed and reported separately according to the particular form of in-patient stroke care. The main outcomes of interest were also analysed and presented in text form according to the various forms of in-patient care interventions and services. Information such as the effect of acute stroke management interventions on key patient outcomes such as in-patient mortality, morbidity and length of hospital stay as well as other variables of interests were assessed. Results were reported in simple statistical or descriptive format comparing patient outcomes across eligible studies. Differences and similarities across interventions were also discussed. In addition, key conclusions of each study were summarised and reported in the evidence table. However, the limited number of included studies, small sample sizes and the heterogeneity of the study outcomes measured made it impossible to conduct a meta-analysis. As a result, the general results were reported as a narrative summary.

## Results

**Fig. 1:**
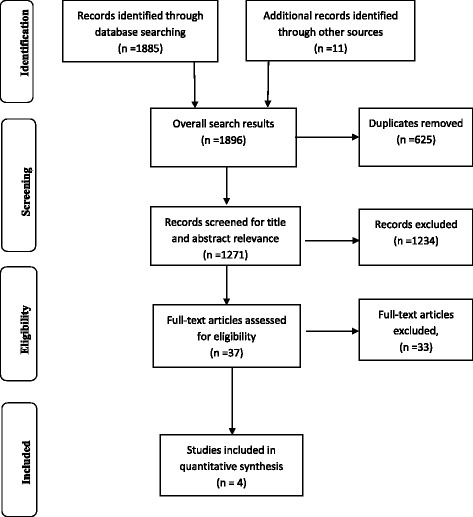
Flow chart on selection of eligible studies

Overall, the search yielded 1896 studies (MEDLINE = 498, CINAHL = 284, Embase = 293, World of Science = 15, Cochrane Library = 147, Academic Search Complete = 648 and 11 from other sources). Of these, 11 studies were from other sources. A total number of 625 duplicates were removed. Another 1234 studies were removed after title and abstract screening for relevance. Consequently, a full-text article screening for eligibility was conducted for 37 studies. The full-text assessment excluded another 33 as they did not meet the eligibility criteria. Finally, a total of four studies met the eligibility criteria for this review. The search results are presented in Fig. 1.

### Characteristics of included studies

A total number of 330 participants were included in this review. Studies were published between 2009 and 2016. Of the four eligible studies, there were no experimental studies; two were retrospective cohort studies [41, 42], a prospective cohort study [43] and a case series study [44]. Three of the studies reported on thrombolytic therapy using recombinant tissue plasminogen activator [42–44] and the remaining study focused on stroke unit care [41]. Three of the eligible studies were conducted in South Africa [41–43] and the other in Morocco [44]. The characteristics of the four eligible studies are summarised in Table 1.

**Table 1:** Characteristics of studies on interventions for acute stroke care

Lead author year and country	Study aim	Study design	Intervention	Level of evidence	Duration	Population sample	Outcome measures	Key results
Villiers et al. 2009 South Africa	Examine the impact of multidisciplinary stroke care on in-hospital mortality, resource utilisation and access to in-patient rehabilitation facilities for stroke patients admitted to the stroke unit	Retrospective study	Stroke unit	Level 3	December 2001–February 2002 March 2002–May 2002	195 patients Mean age = 58.8 60% were female	Length of hospital stay in-patient death transfer to a tertiary hospital number of patients who accessed CT brain	In-hospital mortality was 31 (33%) in general ward compared to 16 (16%) in the stroke unit (*p* = 0.005) Mean length of hospital stay before stroke unit was 5.1 (6.5, 3.8–6.4) days compared with 6.8 (4.5, 5.9–7.6) days after stroke unit care (*p* = 0.01) Access to CT brain scans increased from 13% (12) to 16% (16) Referrals to the tertiary academic hospital 7% (*n* = 7) vs. 4% (*n* = 4) did not change significantly
Wasserman and Bryer 2012 South Africa	To evaluate early outcomes and safety of stroke thrombolysis in a South African setting	Prospective study	Thrombolytic therapy	Level 3	January 2000–February 2011	42 patients	Early neurological recovery functional independence at discharge rate of symptomatic intracranial haemorrhage (SICH) Death	Mean time to t-PA infusion was 160 min (SD 50; range 60–270). 72.5% patients were thrombolysed within 180 min Median NIHSS score fell to 7.5 (IQR 1 to 15) by the time of discharge 67% of patients achieved significant neurological improvement after thrombolysis 40.5% were functionally independent 2 (4·8%) patients suffered SICH 3 (7·1%) patients died at discharge
Klemperer et al. 2014 South Africa	To evaluate the performance of SITS-SICH and SEDAN scores in predicting the risk of SICH after thrombolysis	Retrospective Study	Thrombolytic therapy	Level 3	2000–2012	41 patients	Bleeding complications SICH risk	2 (4.9%) patients experienced SICH, (95% CI: 0–11.5%), SITS-SICH (5.1%) and SEDAN (6.5%) cohorts 23 patients accessed CT brain scan
Naima Chtaou et al. 2016 Morocco	To report the case series of all patients who were treated with rt-PA in a stroke unit of HASSAN II University hospital between 2010 and 2013	Case series	Thrombolytic therapy	Level 4	2010–2013	52 patients mean age = 63 years		17 patients (32.7%) were treated within a 3-h window of stroke onset and 35 (67.3%) within 3–4.5 h 25 patients (48%) had significant early improvements within 24 h, 21 (40.3%) had good outcomes at 3 months and 15 (29%) died Mean door-to-needle time was 75 min and mean onset-to-treatment was 212 min 3 asymptomatic ICH and 4 symptomatic ICHs were reported 2 of the 4 symptomatic ICHs were fatal

### Quality and strength of evidence

On the basis of evidence classification, the three eligible cohort studies were classified as level 3, whereas the case series study fell under level 4 per the Oxford Centre for Evidence-Based Management (OCEBM) levels of evidence for effectiveness [45]. Despite evidence of the efficacy of thrombolytic therapy using t-PA and stroke unit care, the quality of the eligible studies compromised the strength of the evidence. The lack of experimental studies, inadequate measures to account for confounding covariates and absence of randomisation undermined the level of evidence to unequivocally support the effectiveness of thrombolysis and multidisciplinary stroke unit care on clinical outcomes. For the cohort studies, the sampling procedure was only moderately conducted despite those studies employing a clearly defined selection criteria and had reliably measured and analysed patient outcomes.

Despite accounting for confounding factors in the cohort studies, only one study [42] accounted for it in the analysis. Also, the retrospective nature of two of the cohort studies [41, 42], where outcomes were reportedly based on a chart review of medical records, could bias the results by potentially underestimating or overestimating the final outcomes. All included studies contained small and unrepresentative patient samples which limited their generalisability. Methodologically, the case series study, on the other hand, scored highly on the quality appraisal checklist. Nonetheless, it lacked important baseline information, limiting generalizability. On this basis, the overall quality of the cohort studies provides only limited support for the efficacy of such interventions. Quality assessment of included studies is shown in Table 2.

**Table 2:** Risk and quality assessment of eligible studies

	Appraisal questions for cohort studies	Bryer and Wasserman 2012	Villiers et al. 2009	Klemperer et al. 2014
1	Were the groups similar and recruited from the same population?	Yes	Yes	Yes
2	Were the exposures measured similarly to assign people to both exposed and unexposed groups?	Yes	Yes	Yes
3	Was the exposure measured in a valid and reliable way?	Yes	Yes	Yes
4	Were confounding factors identified?	Yes	Yes	Yes
5	Were strategies to deal with confounding factors stated?	No	No	Yes
6	Were the groups/participants free of the outcome at the start of the study (or at the moment of exposure)?	Yes	Yes	Yes
7	Were the outcomes measured in a valid and reliable way?	Yes	Yes	Yes
8	Was the follow up time reported and sufficient to belong enough for outcomes to occur?	Yes	No	Yes
9	Was follow-up complete, and if not, were the reasons to loss to follow-up described and explored?	NA	NA	NA
10	Were strategies to address incomplete follow-up utilised?	NA	NA	NA
11	Was appropriate statistical analysis used?	Yes	Yes	Yes
	Critical appraisal questions for case series study	Naima Chtaou et al. 2016		
1	Were there clear criteria for inclusion in the case series?	Yes	–	–
2	Was the condition measured in a standard, reliable way for all participants included in the case series?	Yes	–	–
3	Were valid methods used for identification of the condition for all participants included in the case series?	Yes	–	–
4	Did the case series have consecutive inclusion of participants?	Yes	–	–
5	Did the case series have complete inclusion of participants?	Yes	–	–
6	Was there clear reporting of the demographics of the participants in the study?	Yes	–	–
7	Was there clear reporting of clinical information of the participants?	Yes	–	–
8	Were the outcomes or follow up results of cases clearly reported?	Yes	–	–
9	Was there clear reporting of the presenting site(s)/clinic(s) demographic information?	No	–	–
10	Was statistical analysis appropriate?	Yes	–	–
11	Were there clear criteria for inclusion in the case series?	Yes	–	–

#### Efficacy of the acute stroke care interventions

##### Stroke unit

One study was identified which reported multidisciplinary team care in a stroke unit [41]. This was a retrospective study to evaluate patient outcomes following multidisciplinary care in a South African stroke unit and a general medical ward among 195 patients. The study outcomes comprised in-patient deaths, patient access to CT brain scan, length of hospital stay and transfer to a tertiary hospital. Overall, the study showed less deaths (16%) in patients treated in the stroke unit compared to the general ward (33%; *p* < 0.005). The mean length of hospital stay prior to the stroke unit was 5.1 days in the general wards, compared to 6.8 days when the stroke unit care was introduced (*p* < 0.01). Stroke patient referrals at discharge to in-patient rehabilitation also increased from 5 to 19% (*p* < 0.04) after introducing the stroke unit. In contrast, only three patients (5%) were referred at discharge for further in-patient rehabilitation in the general wards before the advent of the stroke unit care. Additionally, there was a disparity in access to brain scanning services between the two admitting wards. Patient access to CT brain scan was 12 (13%) in the general medical ward but this increased to 16 (16%) following the introduction of the stroke unit. The difference in access to brain CT scan between the two patient cohorts was not significant.

##### Thrombolytic therapy

Three studies reported on thrombolytic therapy for acute stroke care, two in South Africa [42, 43] and another in Morocco [44]. The first study evaluated outcomes and safety of thrombolysis among 42 patients thrombolysed using t-PA in a tertiary academic hospital [43]. The outcome measures included in-patient deaths, early neurological recovery and rate of symptomatic intracranial haemorrhage (SICH). The results showed 17 (40.5%) participants were being functionally independent at discharge. Risk of bleeding and other complications such as SICH is associated with thrombolysis use globally [17]. This study also found two (4.8%) patients experienced SICH whilst three (7.1%) patients died following thrombolysis.

The second South African study examined the risk outcomes associated with thrombolysis using t-PA among 41 patients [42]. The study outcomes included SICH, deaths, asymptomatic intracranial haemorrhage (AIH) and extracranial haemorrhage (EH). Two instruments were used to predict the risk of SICH; SEDAN and Safe Implementation of Treatment in Stroke (SITS) scores. Overall, the study showed two (4.9%) patients experienced SICH, (95% CI: 0–11.5%) representing 5.1% for SITS-SICH and 6.5% for the SEDAN cohorts. One patient (2.4%) died as a result of SICH following thrombolysis. Evidence of AIH was found in eight (19.5%) patients and another two (4.9%) patients of EH. Of the 41 participants, 23 (56.0%) had access to computed tomography (CT) brain scan prior to the intervention.

The third study, which was a case series, examined patient outcomes following the use of thrombolytic therapy in a stroke unit [44]. Study outcomes measured in this study are deaths, early clinical improvement and clinical morbidities including SICH. The evidence showed that 25 (48%) patients had significant early clinical improvements within 24 h, 21 (40.3%) at 3 months and 15 (29%) in-patient mortality cases. The study reported that the early NIHSS score recorded higher severity in the first patient cohort (NIHSS > 15 in 58% of the patients) compared to the second patient cohort (NIHSS > 15 in 28% of the sub-patient group). The study also noted three (5.7%) asymptomatic intracerebral haemorrhage and four (7.7%) SICH complications. Two of the SICH cases were also fatal.

## Discussion

This study set out to systematically identify the best available evidence on the application of interventions for acute stroke care across hospital settings in Africa. Overall, despite global advancements in best practice interventions for acute stroke care, the evidence base in the African context remains limited. As demonstrated in this review, only four studies were eligible, one evaluating clinical outcomes following stroke unit care and the remaining three on outcomes following thrombolytic therapy. This limited number that met the inclusion criteria highlights the paucity of work on this topic to date. Nonetheless, this limited literature demonstrates improved patient clinical outcomes within the African context. The studies report similar results of improved patient outcomes compared to those studies conducted in other LMIC and HIC settings. Although the limited number of eligible studies, their non-experimental nature and methodological quality issues preclude more definite conclusions, the evidence reported in this review still provides valuable insight towards health policy formulation and future research to optimise clinical management of stroke patients.

### Comparison with previous evidence

Studies that have examined evidence-based acute stroke care interventions in LMICs are scarce. An earlier review on the uptake of thrombolysis in developing countries also found very few studies [29]. A systematic review undertaken by Berkowitz et al., to estimate thrombolytic therapy uptake globally found the use of thrombolytic therapy was 19% in LMICs such as those in Africa [7]. In contrast, uptake was about 50% in HICs. Hence, this current review confirms the previously described paucity of evidence-based acute stroke care interventions in resource-poor settings such as Africa [33].

The reviewed studies confirm previously identified improved patient outcomes in other settings following stroke unit care [16, 46, 47] and thrombolytic therapy [17, 48–50]. This efficacy seems consistent across various country contexts. For example, a reduction of in-patient mortality following stroke unit care in the South African study [41] corroborates with an Indian study, which also found positive patient outcomes following admission in a stroke unit [46]. Further, a Canadian study also found favourable patient outcomes following stroke unit care [51]. This retrospective study compared two community hospitals and found a significant reduction in in-patient deaths (17.1 to 8.3%). This study also found a significant reduction in length of hospital stay from 12 to 8 days. Comparable to the results of the Cochrane review on in-patient care in a stroke unit [16], the present review confirmed lower in-patient deaths in the stroke unit but no reduction in length of hospital stay in the stroke unit.

In this review, three studies reported improved patient outcomes following thrombolytic therapy. The study by Bryer and Wasserman [43] demonstrated a relatively lower SICH and deaths following thrombolytic therapy, consistent with studies in India [52, 53] and Vietnam [48], and some HICs including Western Europe [54] and Australia [55, 56]. Similar positive findings have been reported previously in randomised control trials [17, 18]. In combination, such findings show that thrombolytic therapy can generate optimal patient outcomes in Africa. It is thus imperative for policymakers to increase efforts to upscale the use of thrombolytic therapy in hospital settings to reduce the current disproportionately high stroke burden in Africa. Previous work has identified some potential barriers to the use of thrombolysis in Africa and developing countries in general. According to some authors [33, 57], potential barriers such as patient late arrival for care in a hospital setting, lack of specialist stroke care professionals and inadequate medical facilities such as CT brain scanning services provide specific targets for policymakers.

Although this review did not find studies on aspirin therapy, its low cost and ease of administration [57–59] are likely drivers for its widespread use for acute ischaemic stroke care across hospital settings in Africa. The present review did not find any eligible study on the use of decompressive surgery in the region. However, studies in Nigeria which were excluded because the cases were non-stroke patients did report such intervention [60, 61], suggesting that such interventions may be routine in Africa for acute stroke care but not yet reported in the literature.

### Implications for practice, policy and future research

Stroke is a major public health problem in Africa and current evidence suggest its incidence will rise further. It is therefore important to ensure unimpeded access to standardised acute stroke care in hospital settings. The evidence from this review suggests a likely limited availability of ‘best practice’ interventions for acute stroke care across Africa. The current scarcity of evidence may be due to relatively increased attention on stroke prevention rather than treatment.

As noted previously, although multiple barriers such as limited stroke care specialists, patient delay in seeking care or limited access to brain scanning services may account for the low application of evidence-based acute stroke interventions, the most important barrier may be its cost [33]. This has been demonstrated by a study in Congo where eligible stroke patients could not afford the treatment [62]. Additionally, a feasibility study on thrombolysis provision has been conducted in Senegal [63], suggesting acute stroke patients can be treated with standard acute stroke care in Africa. However, factors such as limited health resources and cost need to be considered.

As there is an urgent global need to translate research evidence to community uptake and policy reform [64], the limited evidence evaluating the effectiveness of stroke unit care in this review requires policy attention. This relatively lower uptake in LMICs compared to HICs may be a function of limited resources, characteristic of most health systems in resource-poor settings. One study which was excluded on the basis of lack of access to a full text reported improved clinical outcomes following multidisciplinary stroke team care in a Nigerian stroke unit [65]. The study reported consistent reductions in annual mortality since the introduction of the stroke unit, thus demonstrating that if more policy support is provided for intervention uptake, improved patient outcomes could be realised.

This review also revealed three studies which confirmed the safety and efficacy of thrombolytic therapy (t-PA). Despite persistent questions about its safety, thrombolytic therapy is recognised internationally as a highly effective pharmacological therapy for acute ischaemic stroke cases. Yet, this review indicated that uptake was limited. Within the context of Africa, resources to support the healthcare systems are often inadequate, affecting access to t-PA which requires administration by specialised stroke physicians and nurses. Patient inability to pay for t-PA [57] and late patient arrival are other major barriers to accessing t-PA [57, 66]. The availability of dedicated stroke units and brain CT scan services facilitate the administration of t-PA and thus are integral to ensuring optimal delivery of t-PA. The availability of decision-making tools, such as the SITS-SICH and SEDAN scores for assessing SICH [42], can support healthcare staff in evaluating the risk-benefit in relation to the selection of eligible patients for t-PA. Thus, the widespread use of such tools should be encouraged.

Overall, the paucity of studies identified in this review suggests a wide evidence-practice gap within the context of acute stroke treatment and management across the African region. Although a recent study illuminated the potential reasons for such underutilisation, it is important to review this within the broader context of the existing constraints to optimal healthcare delivery in Africa. The widespread development and provision of stroke unit care across Africa require major health policy reforms, with consequent budgetary requirements. Within this context, as part of efforts to enhance uptake of contemporary acute stroke care interventions, this review emphasises the need to review the applicability and context appropriateness of current interventions for acute stroke care. This is necessary because the clinical trials which concluded on the efficacy of the current interventions were predominantly from HICs, and thus their applicability to African settings remains unclear. This situation necessitates further research on potential adaptation of such best practice guidelines for resource-poor settings.

Increased patient access to brain imaging services such as CT and MRI scans could optimise the benefits of aspirin towards the treatment of acute stroke and prevention of recurrent stroke. In Africa, access to brain scanning services is limited due to service availability and cost to patients [33]. Subsidies addressing these issues are a potential policy pathway to enhance access and availability. Centralisation of standard acute stroke care services may also be feasible in LMICs as a short-term measure. Centralised stroke care services involve rerouting and transferring suspected stroke cases to a specialist referral centre, often a tertiary hospital and well equipped to provide specialist care. This may replace the current practice of transporting and admitting stroke suspected cases to the nearest hospital. This is important given the underfunded nature of the existing healthcare systems to be able to resource most hospitals with adequate facilities to provide standard acute stroke care. Research in HICs suggests that this method can improve access to standard care for acute stroke patients resulting in reduction in mortality and length of hospital stay [67–70]. Low-cost acute stroke care interventions such as tenecteplase may be a potential alternative and could also be explored due to the high cost of thrombolysis. Although more evidence on the safety and efficacy of tenecteplase is needed, advocating for the use of such cost-effective and low-level evidence interventions could contribute to strategies to minimise the current stroke burden in Africa and other LMIC regions.

The limited eligible studies and low methodological quality of the eligible studies in this review indicates a need for further research, particularly for prospective studies such as randomised control studies, to provide a clearer understanding of the effects of current interventions for acute stroke management on patient outcomes. The limited funds to support such research studies in LMICs such as those in Africa could impede such efforts. In particular, there is a need for information on the factors influencing the low application of such acute stroke care interventions in resource-poor settings such as Africa, particularly from the perspectives of stroke care practitioners, patients, health managers or from health policymakers.

### Study limitations and strengths

The limited number of eligible studies provides only a small evidential base. This may reflect the limited research on evidence-based acute stroke care interventions in Africa. It seems likely that informative research, yet unpublished, is underway currently, for example in Ghana [6], Congo [62], Morocco [44], Nigeria [71] and Egypt [72], indicating further emerging evidence of the availability of stroke unit care in hospital settings. Further, the quality of evidence from the included studies is low as the studies were non-experimental, non-randomised and did not adequately control for confounding covariates. The low ranking of studies in the evidence classification underscores this point. Another limitation worth noting is the small sample size in each of the eligible studies. This inherently compromises the statistical power of the studies to report accurate and precise differences and effects between acute stroke care interventions. Finally, it is possible that the evidence reported in this review may have suffered from publication bias, arising from the publication of only significant results.

Despite the above limitations, to our knowledge, this represents the first systematic review on evidence-based acute stroke interventions and their effects on patient outcomes in the African region. Thus, the findings provide information with the potential to inform health policymakers in developing interventions in the future to optimise patient outcomes.

## Conclusion

Despite the limited studies on current evidence-based acute stroke care interventions in Africa, this review highlights improved patient outcomes, hence the need for policy support to routinise current best practice interventions for acute stroke care. However, because eligible studies were limited and had some methodological weakness, more definitive conclusions require further research which focuses on strong methodological procedures, primarily randomised control trials, to better understand the efficacy of contemporary acute stroke care interventions in the African region.

## Additional file

Additional file 1: Medline search strategy. (DOCX 13 kb)

## Abbreviations

CINAHL: Cumulative Index to Nursing and Allied Health Literature
CT: Computed tomography
HIC: High-income countries
LMIC: Low-middle income countries
OCEBM: Oxford Centre for Evidence-Based Management
PRISMA: Preferred Reporting Items for Systematic Reviews and Meta-Analyses
QASC: Quality in Acute Stroke Care
RCT: Randomised control trials
SICH: Symptomatic intracerebral haemorrhage
t-PA: Tissue plasminogen activator
