# Supplementary material for: Interventions for acute stroke management in Africa: a systematic review of the evidence
Source: Syst Rev. 2017 Oct 24;6:213. doi: 10.1186/s13643-017-0594-4 (PMC5655819; doi:10.1186/s13643-017-0594-4)
Supplement: Additional file 1: — Medline search strategy. (DOCX 13 kb) [file 13643_2017_594_MOESM1_ESM.docx]

**Supplementary File 1: Medline Search Strategy**

| **Search** | **Query** |
| --- | --- |
| 1 | (Stroke) OR (Acute Stroke) OR (Cerebrovascular Disease) OR (Cerebrovascular Accident) OR (CVA) OR (Brain Attack) OR (Cerebral Infarction) OR (Lacunar ) OR (Chronic Stroke ) OR (Neurological Disorder) OR (Brain Tumour) OR (Brain Accident) OR (Brain Vascular Accident) OR (Cerebral Vascular Accident) OR ( Cerebrovascular Trauma) OR (Cerebrovascular Injury ) OR (Cerebral Infarction) |
| 2 | (Stroke unit) OR (Organised Stroke Care) OR (Stroke Ward) OR (Organised In-patient care) OR (Organized Stroke Care) OR (Organised Care) OR (Comprehensive stroke unit) OR (Stroke Service) OR (Rehabilitation Stroke Unit) OR (Multidisciplinary Stroke Care) OR (Multidisciplinary Stroke Team) OR (Stroke Treatment Team) OR (Stroke Management Team) |
| 3 | (Aspirin) OR (Antiplatelet) OR (Fibrinolytic Agents) OR (Acetylsalicylic Acid) OR (Antithrombocytic Agent) |
| 4 | (Thrombolytic Therapy) OR (Intravenous fibrinolysis) OR (Tissue Plasminogen Activator) OR (t-PA) OR (rt-PA) OR (Alteplase ) OR (Thrombolysis) OR (Intravenous IV thrombolysis) OR (Blood Clot Lysis) OR (Fibrinolytic Therapy) |
| 5 | (Decompressive Hemicraniectomy) OR (Craniectomy) OR (Decompressive Surgery) OR (Neuroprotective therapy) OR (Vascular Surgery) OR (Neurosurgery) OR (Decompression Surgery) |
| 6 | (Endovascular Therapy) OR (Endovascular) OR (Thrombectomy) OR (Stent Retriever Thrombectomy) OR (Tenecteplase) OR (Interventional Acute Treatment) OR (Neuro-interventional Management) OR (Brain Treatment ) OR (Brain Management) OR (Acute Stroke Treatment) OR (Stroke Treatment ) OR (Stroke Management) OR (Acute Stroke Management) |
| 7 | (Developing countr*) OR (Low income countr*) OR (Low-middle income countr*) OR (Middle income countr*) OR (Africa) OR (Africa South of the Sahara) OR (Sub-Saharan Africa ) OR (Central Africa) OR (Southern Africa) OR (Northern Africa) OR (Eastern Africa ) OR (Western Sahara) OR (East Africa ) OR (Central African Republic ) OR (West Africa ) OR (Morocco) OR (Libya) OR (Cameroon) OR (Chad) OR (Algeria) OR (Congo) OR (Democratic Republic of Congo) OR (Congo, Demographic Republic) OR (Congo, Republic) OR (Equatorial Guinea) OR (Gabon) OR (Burundi) OR (Djibouti) OR (Eritrea ) OR (Ethiopia ) OR (Egypt) OR (Kenya) OR (Rwanda) OR (Somalia) OR (Sudan) OR (Tanzania) OR (Tunisia) OR (Uganda) OR (Angola) OR (Botswana) OR (Lesotho) OR (Malawi) OR (Mozambique) OR (Namibia) OR (Swaziland) OR (Zambia) OR (Zimbabwe) OR (Benin) OR (Burkina Faso) OR (Cape Verde) OR (Cote D'ivoire) OR (Gambia) OR (Gambia, The) OR (Ghana) OR (Guinea) OR (Guinea-Bissau) OR (Liberia) OR (Mali) OR (Mauritania) OR (Niger) OR (Nigeria) OR (Senegal) OR (Sierra Leone) OR (Togo) OR (South Sudan) OR (Madagascar) OR (Comoros) OR (Mauritius) OR (Sao Tome and Principe) OR (Seychelles) OR (South Africa) |
| 8 | 2 OR 3 OR 4 OR 5 OR 6 |
| 9 | 1 AND 7 AND 8 |
| Limiters | Full Text  English and French Languages  Academic Journals  Humans |
